# Supplementary figures and images for: Development and preliminary validation of the GebStart-tool for advising nulliparous women in early labour
Source: PLoS One. 2025 May 27;20(5):e0322039. doi: 10.1371/journal.pone.0322039 (PMC12112190; doi:10.1371/journal.pone.0322039)

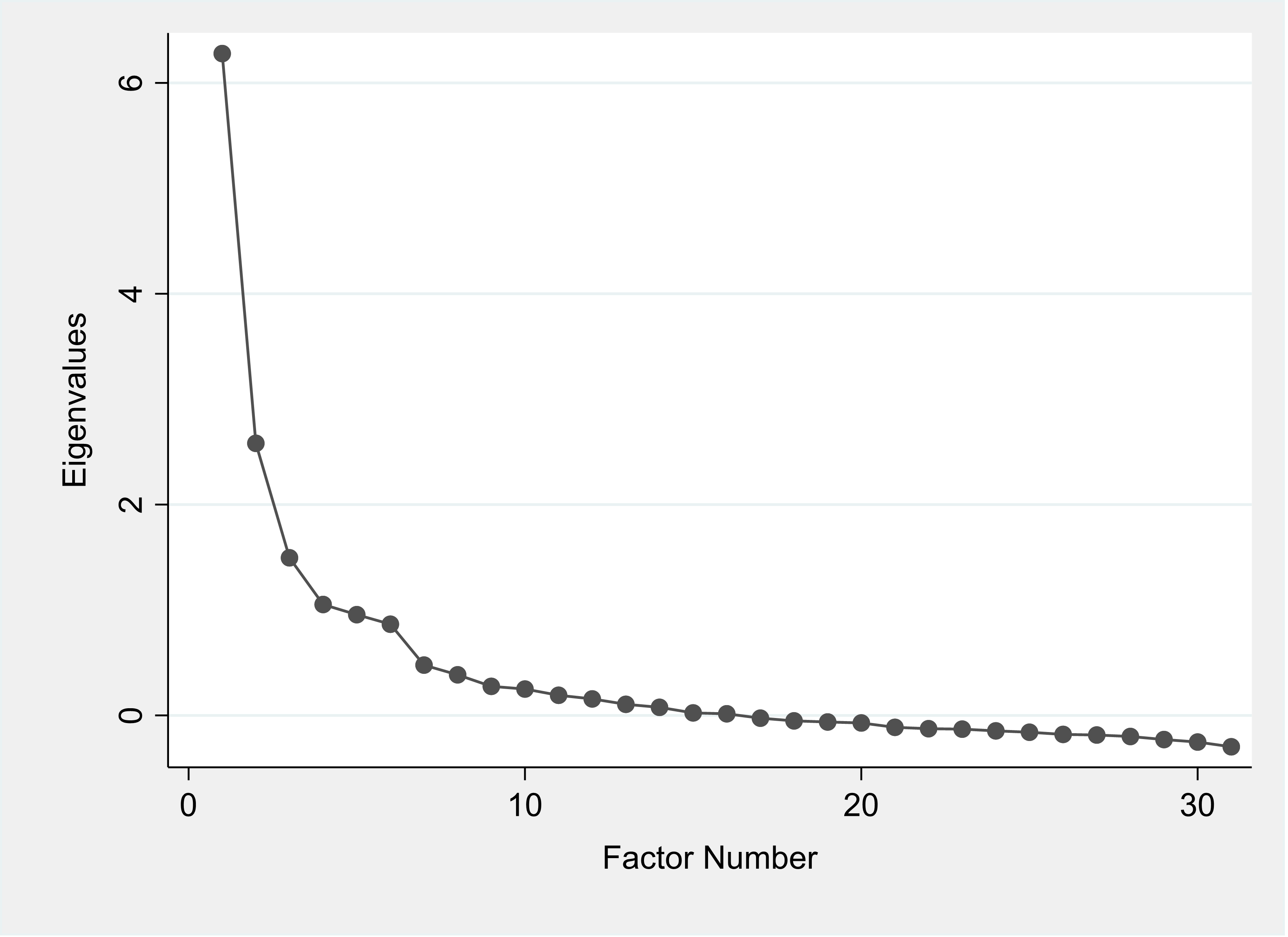

Supplement: S1 Fig — This scree plot supported the decision to subdivide the items into four domains. (TIF) [file pone.0322039.s001.tif]
