# Supplementary material for: Development and preliminary validation of the GebStart-tool for advising nulliparous women in early labour
Source: PLoS One. 2025 May 27;20(5):e0322039. doi: 10.1371/journal.pone.0322039 (PMC12112190; doi:10.1371/journal.pone.0322039)
Supplement: S2 Table — (DOCX) [file pone.0322039.s003.docx]

**S2 Table. Cross loadings (>0.3) of items to the domains “Physical symptoms” “Emotional state” “Self-management” and “Resources” in the PLS-SEM model with the outcomes “Care needs” and “Effective decision”.**

| **Item** | **Aspect, de-scription** | **Physi-cal symp-toms** | **Emotio-nal state** | **Self-ma-nage-ment** | **Re-sour-ces** | **Care needs** | **Effec-tive de-cision** | **Rele-vance** |
| --- | --- | --- | --- | --- | --- | --- | --- | --- |
| Item 1 | Contractions | 0.6710 |  | 0.4434 |  |  |  |  |
| Item 2 | Contractions | 0.6040 |  | 0.3483 |  |  |  |  |
| Item 3 | Contractions | 0.6076 |  | 0.3844 |  |  |  |  |
| Item 4 | Contractions | 0.6967 | 0.3613 | 0.5397 |  |  |  |  |
| Item 5 | Contractions | 0.6609 | 0.3379 | 0.5098 |  |  |  |  |
| Item 6 | Vaginal discharge |  |  |  |  |  | 0.4590 |  |
| Item 7 | Vaginal discharge |  |  |  |  |  | 0.4440 |  |
| Item 8 | Vaginal discharge |  |  |  |  |  |  | Very low |
| Item 9 | Sleep | 0.4437 |  |  |  |  |  |  |
| Item 10 | Fitness | 0.5816 | 0.4283 | 0.3892 |  |  |  |  |
| Item 11 | Fitness | 0.5850 | 0.3677 | 0.3189 |  |  |  |  |
| Item 12 | Gastro-intestinal |  |  |  |  |  |  | Very low |
| Item 14 | Gastro-intestinal | 0.4252 |  |  |  |  |  |  |
| Item 15 | Foetal movement | 0.3170 |  |  |  |  |  |  |
| Item 16 | Foetal movement |  |  |  |  |  |  | Very low |
| Item 17 | Confidence |  | 0.6021 | 0.3173 | 0.3555 |  |  |  |
| Item 18 | Emotional state |  | 0.7194 | 0.3818 | 0.3171 |  |  |  |
| Item 19 | Safe at home | 0.3726 | 0.7456 | 0.5159 |  |  |  |  |
| Item 20 | Worrying |  | 0.6018 |  |  |  |  |  |
| Item 22 | Feeling at home | 0.4863 | 0.7911 | 0.7267 |  |  | 0.3234 |  |
| Item 21 | Reason for call | 0.3984 | 0.5126 | 0.7917 |  |  |  |  |
| Item 23 | Distraction | 0.4782 | 0.4847 | 0.7348 |  |  |  |  |
| Item 24 | Handling | 0.5192 | 0.4629 | 0.7109 |  |  |  |  |
| Item 27 | Preferences management |  | 0.4927 | 0.7011 | 0.3545 |  | 0.4356 |  |
| Item 26 | Attitude |  |  |  | 0.6112 |  |  |  |
| Item 28 | Preparation |  | 0.3116 |  | 0.6213 |  |  |  |
| Item 29 | Support |  |  |  | 0.4430 |  |  |  |
| Item 31 | Companion |  |  |  | 0.4630 |  |  |  |
| Item 32 | Distance to facility |  |  |  | 0.4509 |  |  |  |
| Tool – onset active labour | |  |  |  |  | 0.9857 |  |  |
| Tool – hospital admission | |  |  |  |  | 0.9944 |  |  |
| Tool – pain management^1^ | |  |  |  |  | 0.9953 |  |  |
| Effective decision | |  |  | 0.3399 |  |  | 1.000 |  |

^1^ First medical or alternative pain management
