# Supplementary material for: Development and preliminary validation of the GebStart-tool for advising nulliparous women in early labour
Source: PLoS One. 2025 May 27;20(5):e0322039. doi: 10.1371/journal.pone.0322039 (PMC12112190; doi:10.1371/journal.pone.0322039)
Supplement: S3 Table — (DOCX) [file pone.0322039.s004.docx]

**S3 Table.** **Associations of the total score of the final GebStart-tool with the durations between the completion of the tool and hospital admission, onset of active labour, first medical and alternative pain management.**

| **Predictor** | **Duration tool - hospital admission**  **HR [95% CI]** | **Duration tool – onset of active labour**  **HR [95% CI]** | **Duration tool – first medical pain management**  **HR [95% CI]** | **Duration tool – first alternative pain management**  **HR [95% CI]** |
| --- | --- | --- | --- | --- |
| **Total score GebStart-tool** | 1.08 [1.05,1.10]*** | 1.06 [1.04, 1.08]*** | 1.08 [1.06, 1.11]*** | 1.08 [1.05, 1.10]*** |
| **Age** | 1.02 [0.99, 1.05] | 1.02 [0.99, 1.05] | 1.03 [1.00, 1.07]* | 1.02 [0.99, 1.05] |
| **Nationality Suisse^1^** | 1.09 [0.85, 1.39] | 1.25 [0.98, 1.60] | 1.18 [0.90, 1.55] | 1.22 [0.93, 1.60] |
| **Education university^2^** | 1.16 [0.92, 1.47] | 1.19 [0.95, 1.50] | 1.16 [0.90, 1.51] | 1.29 [1.00, 1.67] |
| **Family income^3^**  Moderate/difficult  Answer declined | 0.64 [0.46, 0.90]**  0.59 [0.26, 1.35] | 0.76 [0.54, 1.05]  0.78 [0.34, 1.76] | 0.48 [0.32, 0.71]***  0.55 [0.22, 1.35] | 0.61 [0.43, 0.87]**  0.66 [0.24, 1.81] |

* p < 0.05

** p < 0.01

*** p < 0.001

^1^ Reference category: Foreign country

^2^ Reference category: Compulsory schooling, vocational training, Matura, higher specialist school

^3^ Reference category: Management with family income good/very good
