# Supplementary material for: Development and preliminary validation of the GebStart-tool for advising nulliparous women in early labour
Source: PLoS One. 2025 May 27;20(5):e0322039. doi: 10.1371/journal.pone.0322039 (PMC12112190; doi:10.1371/journal.pone.0322039)
Supplement: S4 Table — (DOCX) [file pone.0322039.s005.docx]

**S4 Table. Associations of the total score of the final GebStart-tool with the final decision ‘Stay at home – Keep in contact – Hospital admission’.**

| **Predictor** | **Risk ratio** | **95% CI** | **p-value** |
| --- | --- | --- | --- |
| *Decision ‘Stay at home’* | | | |
| Total score GebStart-tool | 0.98 | 0.94-1.02 | 0.421 |
| Age | 0.95 | 0.89-1.01 | 0.125 |
| Nationality Suisse^1^ | 0.84 | 0.48-1.46 | 0.529 |
| Education university^2^ | 0.84 | 0.51-1.36 | 0.477 |
| Family income^3^  Moderate/difficult  Answer declined | 0.64  1.27 | 0.32-1.30  0.20-8.12 | 0.217  0.802 |
| Constant | 10.06 | 0.93-108.82 | 0.057 |
| *Decision ‘Hospital admission’* | | | |
| Total score GebStart-tool | 1.13 | 1.05-1.22 | 0.001 |
| Age | 0.95 | 0.84-1.07 | 0.399 |
| Nationality Suisse^1^ | 0.57 | 0.23-1.41 | 0.225 |
| Education university^2^ | 1.97 | 0.77-5.01 | 0.157 |
| Family income^3^  Moderate/difficult  Answer declined | 0.61  4.78e-06 | 0.16-2.29  0- | 0.460  0.983 |
| Constant | 0.12 | 0.00-10.14 | 0.353 |
| *Reference category ‘Keep in contact’* | | | |

^1^ Reference category: Foreign country

^2^ Reference category: Compulsory schooling, vocational training, Matura, higher specialist school

^3^ Reference category: Management with the family income good/very good
