# Supplementary material for: Development and preliminary validation of the GebStart-tool for advising nulliparous women in early labour
Source: PLoS One. 2025 May 27;20(5):e0322039. doi: 10.1371/journal.pone.0322039 (PMC12112190; doi:10.1371/journal.pone.0322039)
Supplement: S1 File — Final German version of the GebStart-tool after item reduction, including 15 items. (PDF) [file pone.0322039.s006.pdf]

Zürcher Hochschule
für Angewandte Wissenschaften

zh
aw Gesundheit

GebStart

Das GebStart-Tool

Name und Vorname der Frau: ____________________________ Datum und Uhrzeit des Telefonanrufes / Kontakts: ____________________________

Grün: Beobachtung der Fachperson

Gelb: die Gebärende braucht mindestens eine Kontrolle im Krankenhaus

Frage	0	1	2	3	4	Punkte
Wie häufig haben Sie Kontraktionen?	Keine Kontraktionen / hin und wieder	Alle 16-30 Minuten	Alle 11-15 Minuten	Alle 6-10 Minuten	Alle 3-5 Minuten	
Wie schmerzhaft sind die Kontraktionen?	Gar nicht	Ein bisschen	Mittelmässig	Stark	Sehr stark	
Wie verhält sich die Frau während den Wehen?	Keine Wehen / spricht ohne Stocken weiter	Stockt beim Sprechen	Atmet leicht mit	Atmet stark mit	Schreit während den Wehen	
Verlieren Sie Flüssigkeit aus der Vagina und wenn ja, seit wann?	Kein Flüssigkeitsabgang	< 1 Stunde	1-11 Stunden	12–24 Stunden	> 24 Stunden	
Wie beschreiben Sie die vaginalen Abgänge?	Keine / schleimig ohne Blut	Schleimig mit Altblut	Schleimig mit wenig Blut	Flüssig, klar, eventuell mit wenig Blut	Starke Blutung oder grünliche Flüssigkeit	
Fühlen Sie sich fit?	Sehr fit	Eher fit	Mittelmässig	Eher erschöpft	Sehr erschöpft	
Wann haben Sie das letzte Mal gegessen?	Erst gerade	Vor ein paar Stunden	Innerhalb der letzten 12 Stunden	Innerhalb der letzten 24 Stunden	Nicht mehr seit > 24 Stunden	
Spüren Sie Ihr Kind sich bewegen?	Sehr viel	Eher viel	Mittelmässig	Eher wenig	Sehr wenig, gar nicht	

Zürcher Hochschule für Angewandte Wissenschaften

zh aw Gesundheit

GebStart

Frage	0	1	2	3	4	Punkte
Wie zuversichtlich sind Sie für die bevorstehende Geburt?	Sehr zuversicht-lich	Eher zuversicht-lich	Mittelmässig	Eher nicht zuver-sichtlich	Gar nicht zuver-sichtlich	
Wie fühlen Sie sich zu Hause?	Wohl, möchte gerne noch zu Hause bleiben	Eher wohl, kann sich vorstellen, noch zu Hause zu bleiben	Mittelmässig wohl, unsicher, ob zu sie zu Hause bleiben möchte	Eher unwohl, wäre froh, nicht zu Hause bleiben zu müssen	Unwohl, möchte auf keinen Fall zu Hause bleiben	
Wie können Sie mit den Wehen umgehen?	Sehr gut / keine Wehen	Eher gut	Mittelmässig	Eher nicht gut	Gar nicht gut	
Fühlen Sie sich gut auf die Geburt vorbereitet?	Sehr gut	Eher gut	Mittelmässig	Eher nicht gut	Gar nicht gut	
Haben Sie Unterstützung zu Hause?	Sehr viel Unter-stützung	Eher viel Unter-stützung	Mässig viel Unter-stützung	Eher wenig Unter-stützung	Keine Unterstüt-zung	
Wie gut kann Ihre Begleitperson mit der Situation umgehen?	Sehr gut / nicht beurteilbar	Eher gut	Mittelmässig	Eher nicht gut	Gar nicht gut	
Wie viel Zeit brauchen Sie, um das Krankenhaus / Geburtshaus zu erreichen?	< 10 Minuten	10-29 Minuten	30-44 Minuten	45-60 Minuten	> 60 Minuten	
**Total Punkte**						

< 22 Punkte: Zu Hause bleiben
22 - 33 Punkte: Beobachten: entweder ein Folgetelefonat oder eine Kontrolle im Krankenhaus / Geburtshaus vereinbaren
> 33 Punkte: Ins Krankenhaus / Geburtshaus eintreten

Gemeinsamer Entscheid mit der Gebärenden (bitte Zutreffendes ankreuzen):

Bleibt zu Hause und meldet sich bei Bedarf wieder	
Bleibt zu Hause, Telefontermin vereinbart	
Kontrolle im Krankenhaus / Geburtshaus vereinbart	
Eintritt ins Krankenhaus / Geburtshaus	
