# Supplementary material for: Development and preliminary validation of the GebStart-tool for advising nulliparous women in early labour
Source: PLoS One. 2025 May 27;20(5):e0322039. doi: 10.1371/journal.pone.0322039 (PMC12112190; doi:10.1371/journal.pone.0322039)
Supplement: S2 File — The GebStart-tool, preliminary, not validated version. (PDF) [file pone.0322039.s007.pdf]

Preliminary, not validated translation

The GebStart-Tool

Name and surname of the parturient: ______________________ Date and time of phone call / contact: __________________________

Green: Observation by health professional Yellow: the parturient needs at least a check-up in the hospital

Question	0	1	2	3	4	Points
How often do you have contractions?	No contractions / occasionally	Every 16-30 minutes	Every 11-15 minutes	Every 6-10 minutes	Every 3-5 minutes	
How painful are the contractions?	Not at all painful	A little painful	Moderately painful	Rather painful	Very painful	
How does the woman behave during contractions?	No contractions / continues to speak without stopping	Stops when speaking	Breathes lightly during the contractions	Breathes heavily during the contractions	Screams during the contractions	
Do you lose vaginal fluid and if so, since when?	No fluid discharge	< 1 hour	1-11 hours	12–24 hours	> 24 hours	
How do you describe the vaginal discharge?	None / slimy without blood	Slimy with old blood	Slimy with little blood	Liquid, clear, possibly with little blood	Severe bleeding or greenish fluid	
Do you feel fit?	Very fit	Rather fit	Moderately	Rather exhausted	Very exhausted	
When did you last eat?	Just now	A few hours ago	Within the last 12 hours	Within the last 24 hours	Not for > 24 hours	
Do you feel your baby move?	Very often	Rather often	Moderately	Rather rarely	Very rarely/ never	

Preliminary, not validated translation

Question	0	1	2	3	4	Points
How confident are you about the upcoming childbirth?	Very confident	Rather confident	Moderately	Rather not confident	Not confident at all	
How do you feel at home?	Well, wants to stay at home	Rather well, can imagine staying at home	Moderately well, unsure whether she wants to stay at home	Rather unwell, would be happy not to have to stay at home	Unwell, definitely does not want to stay at home	
How can you cope with the contractions?	Very well / no contractions	Rather well	Moderately	Rather not well	Not well at all	
Do you feel well prepared for childbirth?	Very well	Rather well	Moderately	Rather not well	Not well at all	
Are you supported at home?	Very well	Rather well	Moderately	Rather not well	Not well at all	
How well can your accompanying person cope with the situation?	Very well / not applicable	Rather well	Moderately	Rather not well	Not well at all	
How much time do you need to reach the hospital / birthing centre?	< 10 minutes	10-29 minutes	30-44 minutes	45-60 minutes	> 60 minutes	
**Total Points**						

Preliminary, not validated translation

< 22 Points: Stay at home
22 - 33 Points: Observation: either arrange a follow-up telephone call or a check-up at the hospital / birthing centre
> 33 Points: Hospital / birthing centre admission

Joint decision with the parturient (please tick the appropriate box):

Stays at home and reports back when needed	
Stays at home, telephone appointment arranged	
Check-up at the hospital / birthing centre agreed	
Admission to hospital / birthing centre	
