# Supplementary material for: Development and preliminary validation of the GebStart-tool for advising nulliparous women in early labour
Source: PLoS One. 2025 May 27;20(5):e0322039. doi: 10.1371/journal.pone.0322039 (PMC12112190; doi:10.1371/journal.pone.0322039)
Supplement: Attachment — Submitted filename: Revision_Response to editor and reviewer_ GebStart-tool.pdf [file pone.0322039.s010.pdf]

Response to the editor und the reviewer

Editorial comments

Comment	Responses/adaptations
1. Please ensure that your manuscript meets PLOS ONE's style requirements, including those for file naming.	Thank you very much for this comment and the links. We adjusted the manu-script, tables, figures and supporting in-formation accordingly.
2. PLOS ONE has specific requirements for studies that are presenting a new method or tool: the questionnaire or scale must be openly available under a license no more restrictive than CC BY. In light of this, before we proceed, please include a copy of your questionnaire or scale as a Supporting Information file or provide a link if it is available through an online re-pository.	We are happy to add the definitive Ger-man version of the GebStart-tool as Sup-porting Information and referenced it in the text:<br>S1 File. Das GebStart-Tool.<br><br>As the current version of the tool was de-signed to complete by the health care professional together with the parturient, we added on lines 359-361:<br><br>*“The final German version of the GebStart-tool (S1 File) was designed to be completed by health professionals to-gether with the parturients.”*
3. We note that the grant information you provided in the ‘Funding Information’ and ‘Financial Disclosure’ sections do not match.	Many thanks for spotting this discrepancy which we were happy to correct.
4. Thank you for stating the following finan-cial disclosure:<br>"This study was funded by the Swiss Na-tional Science Foundation with a Practice-to-Science Grant (PT00P1_199085)."<br><br>Please state what role the funders took in the study. If the funders had no role, please state: "The funders had no role in study design, data collection and analy-sis, decision to publish, or preparation of the manuscript."	Thank you for proposing this amendment, which was added on lines 489-490 in the manuscript (with track-changes)
5. In the online submission form, you indi-cated that your data will be submitted to a repository upon acceptance. We strongly recommend all authors deposit their data before acceptance, as the process can be lengthy and hold up publication timelines. Please note that, though access re-strictions are acceptable now, your entire minimal dataset will need to be made	We are happy to share the data from the GebStart-Study simultaneously with the publication of this article. Therefore, we prepared and uploaded it with the related metafiles (code book and data processing information) to Zenodo (https://ze-nodo.org/). We uploaded all variables un-derlying this publication. Because of data protection, study site specific study IDs

freely accessible if your manuscript is accepted for publication.	were replaced with consecutive numbers. All dates were omitted but the calculated time intervals were included in the data set. Additionally, some free text comments were deleted to remove information that could indicate persons or locations. Few variables (care models, Mother-Generated Index) were removed because of planned further publications and because data was not used for this publication. Data from n=28 participants of which n=10 had a completed tool, had to be removed because these women did not give consent for further data use. The data uploaded on Zenodo received the following doi-number: 0.5281/zenodo.12773347. Once the manuscript will be accepted, data will be openly accessible immediately.<br><br>We amended the manuscript as follows on lines 499-500:<br><br>*"Data was uploaded to Zenodo and is available at the following link: 0.5281/zenodo.12773347."*
6. Your ethics statement should only appear in the Methods section of your manuscript. If your ethics statement is written in any section besides the Methods, please delete it from any other section.	We are happy the delete the separate ethics statement at the end of the manuscript.
7. Please include captions for your Supporting Information files at the end of your manuscript, and update any in-text citations to match accordingly. Please see our Supporting Information guidelines for more information	Many thanks for this request. We added the captions in the Supporting Information paragraph on lines 462 and following updated as well the in text-citations.

Reviewer #1

Comment	Responses/adaptations
1. Introduction: I'm a little challenged by the overall premise of the model. You report that patients should remain at home as long as possible to avoid unnecessary labor interventions, but for many patients laboring at home without sufficient support is unsatisfying. This is presented as a dichotomy, but could we improve the process of labor care such that we can support people in early labor without a decision tool?	Many thanks for this suggestion. We are happy to provide additionally explanations to describe the need of the study. We added on lines 67-72 (manuscript with track changes) following sentences and adapted the subsequent sentence:<br><br>*"However, especially in times of high workload and financial pressure, advices might depend on the circumstances and can be arbitrary [11,12]. In addition, staying at home over 24 hours in women with prolonged early labour was also found to be associated with negative birth outcomes and an increased risk for caesarean section [13]. Therefore, this well-intended act to delay hospital admission…"*<br><br>Furthermore, we added on lines 84-85 to improve the justification of the study:<br><br>*"There is a lack of tools to assist early labour assessment in an evidence-based way to avoid arbitrary recommendations."*
2. Methods - can you please clarify planned sample size? For example you write that the target sample size was 550 participants assuming 20% dropout rate but that would target an analytic goal of 440 patients and I don't see in the paragraph where that number is explained.	We are grateful that you spotted this inconsistency. The estimated drop out was approx. 25% and not 20% as described in the study protocol. We then rounded the target sample size up to n=550 participants. This was amended on lines 112-117.
3. I recognize the desire to maximize sample size, but given your tool is designed to determine which patients should present to the hospital inclusion of the patients who are in active labor is problematic with the adjustment in time to 0.001 hours. At very least, I think you need a sensitivity analysis re: stability of your results to this assumption.	Thank you for this comment. All time points were assessed with minute precision and time intervals were therefore calculated also with minute precisions. Three decimals were only used in S2 Table for cross loadings in the PLS-SEM models. However, we agree that it is a limitation of the study that some women might have been in active labour at the time of completing the tool. We therefore added in a further limitation on lines 439-441:

	"Additionally, some women with advanced cervical dilatation at hospital admission might have been in active labour at the time of completing the GebStart-tool."<br><br>Additionally, we performed sensitivity analysis for the validation of the study excluding participants with cervical dilatations >6cm at hospital admission. The World Health Organization proposed the end of early labour at 5cm of cervical dilatation. As the GebStart-tool was mostly completed through telephone contact before hospital admission and because cervical dilatation could not be estimated at this time, 6cm appeared to be a reasonable threshold. We conducted a sensitivity analysis for the validation of the tool omitting all women with cervical dilatation >6cm at hospital admission. We added in the methods section the following paragraph on lines 201-208:<br><br>*"As some women were admitted to the hospital in active labour, sensitivity analysis was performed for the Cox regression models, the multinominal regressions, as well as the Generalised estimating equation (GEE) models used for the validation excluding women with cervical dilatation >6cm at the first vaginal examination after hospital admission. The threshold of 6cm was decided because the World Health Organisation (WHO) defined the end of early labour at 5cm [4] and the cervical dilatation could not be assessed at the time of the completion of the tool. The median time interval between the first completion of the tool and hospital admission lasted 3.5 hours."*<br><br>In the result subchapter for the Validation (lines 316 and following), we referred to the sensitivity analysis several times.
4. Results - can you clarify what is meant by "median workload"? Is this equivalent	Thank you for this question. In Switzerland, people very often work part time.

to saying someone is a full time employee rather than someone who works a part time position?	For this reason, it seems important to indicate the median of workload percentages. A 100% workload corresponds to a full-time employment. We are happy to add a further footnote to Table 1:<br><br>*"[3] A 100% workload corresponds to full-time employment and lower percentages to a part time position"*
5. I am not certain we can identify the correct threshold for hospital admission based on figure 3, since the percentage of patients recommended to 'keep in contact' is still higher.	We agree that the second cut-off point is not self-explanatory, because there is no intersect between the curves on the graph. However, the cut-off points could also be calculated according to the suggestions of Bersabé & Riva (2010). We added this explanation to the heading of the figures. Additionally, we amended the explanations for the graph in the text on lines 349-350:<br><br>*"However, for the second cut-off point, the plotted curves did not intersect, even though the values could be calculated according to the suggestions by Bersabé & Riva [32].."*<br><br>And finally, we added a further limitation on lines 450-453:<br><br>*"And finally, it must be noted that the second cut-off point for the decision 'Hospital admission' could be calculated but was not visible in the graph plotting the expected probabilities for the final decision categories with respect to the total score of the GebStart-tool."*
6. Discussion: As a practicing clinician, I was left wondering about the role of this tool. The tool seems to perform pretty modestly at predicting admission / etc, and requires significant effort to complete as you note. I can imagine if I were to tell a patient that they should remain at home or come to the hospital because that's what most pregnant people chose to do under similar circumstances people re-	Thank you for pointing out the missing discussion of the usefulness of the GebStart-tool for clinical practice. We are happy to add a paragraph in the discussion on lines 420 and following:<br><br>*"The significant reduction from 32 to 15 items for the final GebStart-tool enabled to provide a much more manageable tool for clinical practice than it was possible*

sponding that this is a case in which direct elicitation of individual preferences and shared decision making would likely be faster and more fruitful.	*during the study situation. Nevertheless, many aspects were retained, allowing the decision for or against hospitalisation to depend on more than just contractions and vaginal discharge. This is important, because yet some pregnant women in early labour have individual needs and complain about unsatisfactory care and arbitrary decisions [1,10–12,25]. The application of the GebStart-tool has the potential to support shared decision-making about the best place to stay for every induvial parturient. However, further research is necessary to investigate the application of the shortened tool and its benefits."*
