# Supplementary material for: Development and preliminary validation of the GebStart-tool for advising nulliparous women in early labour
Source: PLoS One. 2025 May 27;20(5):e0322039. doi: 10.1371/journal.pone.0322039 (PMC12112190; doi:10.1371/journal.pone.0322039)
Supplement: Attachment — Submitted filename: Revision2_Response to the reviewers_ GebStart-tool.pdf [file pone.0322039.s011.pdf]

Response to the reviewers revision 2

Reviewer #1

Comment	Responses/adaptations
1. I appreciate the detailed response to my prior comments and have no further comments at this time.	Thank you very much for your positive comment.

Reviewer #2

Comment	Responses/adaptations
1. Title:<br>These are not primiparous women – see below<br><br>Same for the abstract	Many thanks for this comment. The terminology is clear to us. We used "primiparous" because, in clinical practice, it is commonly applied to women giving birth to their first child once labour has begun. However, we are happy to replace "primiparous" with "nulliparous" throughout the manuscript if preferred.
2. In the abstract conclusion I think that the term 'evidence-based' is somewhat confusing and disingenuous. The development methodology may be so, but using this term indicates that the tool itself has been the subject of a prospective interventional study or part of a systematic review, and this is clearly not case. Either change the phrase to be more precise or remove it entirely.	Thank you for this comment. We are happy to rephase the sentence as follows:<br><br>Line 47-48: *"We developed a practical instrument with 15 items based on scientific evidence."*
3. Introduction:<br>I agree with the rational for an objective early labour assessment tool and the issues related to early or late presentation at the hospital.<br>This section is well written and creates a cogent and coherent argument for the study.	Many thanks for the positive feedback to the introduction
4. Methods:<br>Typo : line 95 hospitals (plural)	Thank you for spotting this typo, which was corrected.
5. Terminology issues: primiparous versus nulliparous	As mentioned above, "primiparous" was replaced with "nulliparous" throughout the whole manuscript.
6. Very difficult to understand what the plan was and how it was executed. Unlike as it written in the BMJ Open protocol, it is unclear at which point and how the tool was implemented; and by whom. I suggest that this information is added as written in the published protocol (see below)	Many thanks for this important comment. We organised the subchapters in this part of the methods into "Recruitment and data collection" and "Ethics" and added the following sentence to describe the data collection:<br><br>Line 141 and following: *"The preliminary tool, consisting of 32 items for structured*

	assessment, was applied during the first contact with the parturient in early labour, whether this occurred via telephone or in person. Midwives administered the questionnaire by posing the questions verbally. If women called repeatedly, the tool could be utilised multiple times.”
7. I understand why the CBSEI and the CWS were used to validate the new tool, but it seems that neither has an exceptionally high sensitivity for objectively assessing confidence to cope with labour and nor accurately evaluating concerns about labour. Were there no other options? Also, it states that German language versions were used but were these translated versions also correctly validated prior to inclusion in the study? Each newly translated questionnaire requires validation prior to clinical use.	Thank you for these questions. The nature of the GebStart-tool differs largely from other instruments. Therefore, there was no Gold-standard of an instrument for assessing convergent validity for a larger part of our instrument. Regarding Childbirth self-efficacy, there is no other instrument available and the Cambridge Worry scale was widely used in other studies. We used validated German versions of the instrument and added the term *“validated”* in the methods section.
8. These questions are very important as these methods were fundamental to the validation of the GebStart tool.	As we included many different aspects in the reduced GebStart-tool and finally only two items about the emotional state, assessing convergent validity was much less important than previously assumed. We agree that these questions are important, but probably less important than for psychometric scale. However, we are happy to add in the limitation section:<br>- Line 449-450: “*Future studies could explore other scales, such as those assessing fear of childbirth.”*
9. Line 127: typo – mandatory (missing y)	Thank you, this was adjusted.
10. Line 129: ‘accidentally’ only the first subscale – this means what exactly? Full explanation in the text please.	We intentionally used only the labour subscale of the CBSEI and included the 15 items presented in the publication of the German version by Schmidt et al. 2015, see also comment below. We specified this in the methods section and amended the limitation:<br>- Line 130 and following: *“…, the validated German versions of the Childbirth Self-Efficacy Inventory (CBSEI, 15 labour items published by Schmidt et al.2015)…”*

11. Line 164: can you do this and keep the data relevant? The Cox Regression excludes negative time intervals for good reason.	It seems very important not to lose women with advanced labour, because in clinical practice, results of the applied tool are relevant for them. Together with the statistician it was decided that the impact of this adjustment was less than the exclusion of these women.
12. Line 177: I find the term 'outcome effective management unclear. Could this be changed or phrased better?	We agree that the term "effective management decision" is difficult to understand and are happy to change it throughout the manuscript into *"Care decision"*.
13. Do the authors know, on average, how long it took for the study midwives/clinicians to complete the 32-item questionnaire?<br>Someone who is anxious, and in discomfort is probably not going to want to spend too much time completing a questionnaire about their current condition. They want a fairly rapid response to their question: stay at home or come to hospital.<br>Presumably a 15-item questionnaire would take about half as long to get through.	We received feedback indicating that 32 questions were perceived as too comprehensive, taking approximately 15 minutes to complete. However, we also heard from the study midwives at the participating centres that some women greatly appreciated the time spent speaking with them. This interaction often resulted in the women feeling more confident and opting to remain at home. Early labour is frequently a quite long phase, during which women, if labour has not yet progressed significantly, express a desire to engage in conversations with healthcare professionals. We think that the 15-item tool has now an adequate length. In more urgent cases, it becomes quickly clear that women need professional care, and the completion can be canceled.<br><br>The challenging with applying a long preliminary tool was also investigated during this study and was recently published. We referenced this publication in the manuscript:<br>Mueller AN, Grylka-Baeschlin S. The application of a tool for supporting shared decision making in primiparous women during early labour - a descriptive survey of obstetric health care professionals' opinion. BMC Health Serv Res. 2024 Dec 21;24(1):1633. doi: 10.1186/s12913-024-12096-4. PMID: 39709387; PMCID: PMC11662463.
Results:<br>14. Line 216 The term dropouts (37%) suggest a voluntary withdrawal from the	Thank you for highlighting this important point. We replace "dropout" with "excluded", also in the methods section and

study, however the context in which this is used is more of an exclusion. For example, a woman who was induced or underwent caesarean before the onset of labour. These were recruited women who were EXCLUDED but they were not 'dropouts' in the classic sense<br>For 15% of the 37% of dropouts (92 of 233) it is stated that the tool was not completed: reasons = forgotten, too much work or admission was urgent. BUT in the protocol, the tool was to be applied at the onset of labour, so for planned inductions and caesareans before the onset of labour, these patients did not satisfy the inclusion criteria to complete the GebStart Tool. Correct? So how could it have been forgotten when the patients were never suitable?	in the header of Table 1. Additionally, we explained in more details that there were some women with onset of labour for whom there was no time or it was forgotten to complete the tool. We are happy to explain this in more details:<br><br>Line 222 and following*: "Since women were recruited during prengnancy a high number of women had to be excluded by the time they gave birth (n=233, 37.2%) due to either medical induction, caesarean section before onset of labour or in some women, because, the GebStart-tool was not completed, even though they had a spontaneous onset of labour. Reasons for the latter cases (n=95, 15.2%) were that the workload in the study sites was high, hospital admission was urgent or completing the tool was forgotten."*
15. Characteristics of the population<br>The population is highly selective and would not be representative of populations in other similar European countries in terms of marital status, educational attainment, full-time employment, financial status, medical insurance and antenatal preparation. This is not a typical large (university) hospital population, so the results are unlikely to be externally valid. These characteristics are also likely to suggest better physical and psychological preparation and coping strategies for labour, which may produce results considered as outliers when compared to more standard maternity populations.	Thank you for pointing out this important aspect. The high educational level is a problem in most studies. Other aspects might also differ from country to country. We are happy to add a further limitation:<br><br>Line 439 and following: *"Additionally, the high education level of participants, coupled with other socio-demographic factors and the recruitment of women interested in early labour, limits the generalisability of findings."*
16. Line 231 cannot be correct. If all participants were primiparous (one previous liveborn) how could 82% be in their first pregnancy? (primigravid) The terminology is incorrect throughout and must be amended. You cannot be both primiparous AND primigravid. This means that the terminology must be consistent throughout the article.<br>Primigravid or nulliparous but not primip-	As mentioned above, "primiparous" was consequently replaced with "nulliparous" and the sentence slightly adapted (deletion of the word "also").

arous. Please amend to provide consistency in the article.<br><br>Participants have been defined as 'women expecting their first child'.<br>So nulliparous OR primigravid please but not primiparous.	
17. Item reduction<br>This section is quite clear and well written despite being a rather complex process	Thank you very much for this positive comment
18. Validation<br>Should not the validation of the FINAL tool come after the section on cut-off points and designing the final versions? This is a bit confusing made more so by the terminology and use of final Gebstart-tool for two different time points.<br>Is the final GebStart-tool mentioned in the validation section Line 310-311, the same final Gebstart-tool mentioned in Lines 351-360? I believe that this cannot be true.	Thank you for this suggestion. We are happy to place the validation chapter after "Cut-off points and "Designing the final version of the tool". Additionally, "Validation" was replaced with "*Preliminary validation*" in the title but also throughout the manuscript.<br>Regarding your question for the final GebStart-tool: the analyses for the preliminary validation were done with the remaining 15 items of the preliminary GebStart-tool. An explanation was added at the beginning of the preliminary validation chapter.
19. Validation was a mathematical/comparative validation (using amongst other the CBSEI + CWS scores) rather than a prospective clinical validation of the final tool. I assume that there is a plan to prospectively evaluate the FINAL tool in clinically diverse populations, to confirm or refute the findings.<br>Could you also explain the difference between full CBSEI scale and a partial scale? Is a partial scale as valid as a full one? Please clarify in the text as this was an error it seems. And does this not weaken the assessment of the validation?	This is correct, it was a mathematical validation and we changed "Validation" into "Preliminary validation". Additionally, we now use "reduced tool" for the analysis with the 15 remaining items and final tool for the slightly adapted final version.<br>We intentionally used only the labour subscale of the CBSEI and used the 15 items presented in the publication of the German version by Schmidt et al. We specified this in the methods section and amended the limitation:<br>- Line 130 and following: *"…, the validated German versions of the Childbirth Self-Efficacy Inventory (CBSEI, 15 labour items published by Schmidt et al.)…"*<br>- Line 446 and following: "*Another limitation was the use the shortened CBSEI scale (15 items) published by Smidt et al. [27]. Although its reduction has been debated in the literature [27] the impact remains unclear..*"

20. Cut-off points for the final instrument<br>The cut-off points are explained and the lack of intersect for the second-one, justified by the Bersabé and Riva reference. However, I think that given this anomaly, and the importance of the cut-off to the clinical use of the tool, the authors need to give more detail as to why they feel that this calculation (statistical fudge) is valid in these circumstances. Just citing reference is insufficient for me.<br>This is discussed in the strengths and limitations, but its significance is not explained.	We used to different methods for calculating the cut-off points and decided to show results from both for transparency reasons. The calculated method according to Bersabé and Riva provided a clear result. However, the second point was not confirmed by the plot. We added following sentence to this subchapter:<br><br>Line 323 and following: *"In order not to keeping women from receiving care and for safety reasons, the calculated second cut-off point oat 33 was retained."*
21. Designing the final version<br>Just to be sure, it was not this final version with15 items and described modifications that was prospectively trialed clinically, rather the full 32 item list that was then reduced. This was more of a mathematical validation based on the patient responses to the 15 items selected from the original 32, aided by the CWS and partial CBSEI scoring systems to provide some alternative indirect clinical validity. It seems that if the items were re-phrased based on the feedback of study centers, then the true final version of GenStart-tool is yet to be trialled/studied?<br>This is not very clear and needs to be clarified	The 32 items were applied clinically and yes, it was a preliminary, mathematically validation of the tool. The adaptations of the items were minimal. We addressed your concern as follows:<br>- "Validation" was replaced with "preliminary validation" throughout the manuscript.<br>- A first sentence was added to this subchapter:<br>Line 327: "*The final version of the GebStart-tool was designed by using the remaining 15 items."*<br>- In the conclusion, the amended the following sentence:<br>Line 458-459: *"Further research is necessary for definitive validation of the GebStart-tool in larger and more divers samples."*
22. Discussion:<br>Line 364 – primigravid NOT primiparous.	See above, this "primiparous" was changed into nulliparous throughout the whole manuscript.
23. I would rephrase this whole paragraph as the it is the most important at summarising the findings of the study. In this form, the findings are not very clear.<br>'Effective management decision' – this term is a quite ambiguous in isolation. Why effective and not just management decision? Stay at home, stay in contact or go to hospital?<br>The output of the study was indeed a short GebStart tool but to state at this	Thank you for pointing out the weakness of the first paragraph of the discussion, which was rephrased as requested.

stage, without real world evaluations in diverse populations, that it is practical, remains to be confirmed.	
24. Line 417-418: The results OF not TO the GebStart-tool should demonstrate this? Does this mean demonstrated in future clinical studies?	Thank you for this concern. We deleted this sentence.
25. Strengths and weaknesses<br>I am not sure that the readers will correctly interpret 'evidence-based' as described. I assume that the authors refer to an 'evidence-based methodology' and nothing else.<br>They need to be very careful referring to 'evidence-based' as many will assume that this relates to the clinical use of the tool rather than its development. It would be quite a stretch to consider the GebStart tool 'evidence-based at such an early stage of development.	Many thanks for this comment. We changed the first sentence as follows:<br><br>Line 432-433: *"A key strength of this study was the development of the GebStart-tool based on scientific evidence tailored to its purpose."*
26. Also, the study population is certainly not like that of any hospital where I have ever worked, and I have been lucky enough to have worked in several countries on 3 different continents. This makes external validity of the tool in other regions/countries essential if you are to prove its effectiveness. Currently it seems that it is designed for wealthy, well-educated, well insured and well supported married women. This type of population in most other countries is only found in the private sector.<br>These women are more likely to be better prepared for birth, physically and mentally stronger than most women that are seen in other larger European units.<br>As the tool attempts to evaluate coping and confidence in early labour, I would argue that the results of this study are only valid for this specific and unique group of women.	As mentioned above, we are happy to add a further limitation, see also comment 15 and comment from the other reviewer below.<br><br>Line 439 and following: *"Additionally, the high education level of participants, coupled with other socio-demographic factors and the recruitment of women interested in early labour, limits the generalisability of findings"*
27. The discussion is too long and as with other sections, is not easy to follow. I would suggest a complete re-write and then ask for feedback from some native	Thank you for this concern. We edited the discussion and simplified and shortened the text thoroughly.

English speakers, to confirm that it is relatively easy to understand.	
28. I agree with the authors that more work is needed. At this stage the tool has been implemented in very small numbers in a very specific population of pregnant women, who are not at all representative of most women attending larger, university maternities in other European countries.	Many thanks. We are happy to amend this sentence in accordance to the additional limitation:<br><br>Line 458-459: *"Further research is necessary for definitive validation of the GebStart-tool in larger and more divers samples."*

Reviewer #3

Comment	Responses/adaptations
1. The aim of the study is interesting. The scientific answer is rather well constructed, with a coherent statistical basis. But I wonder about the selection bias, which I think reduces the external validity of this study. Indeed, patients who agree to be included are less worried about staying at home, which is not necessarily the case for a large part of the population. This type of patient will not come to hospital if the midwife is reassured that she is not in labour.	Thank you very much for your positive feedback. We agree that research often attracts participants with an interest in the topic. In this study, however, potential participants were informed that the tool aimed to support identifying both women who could safely remain at home and those requiring additional care. This may also have attracted women concerned about not being taken seriously by hospital staff. We are happy to include an additional limitation regarding the study sample, as noted in the comments from the other reviewer:<br><br>Line 438 and following: *"Additionally, the high education level of participants, coupled with other socio-demographic factors and the recruitment of women interested in early labour, limits the generalisability of findings"*
