# Supplementary material for: Development and preliminary validation of the GebStart-tool for advising nulliparous women in early labour
Source: PLoS One. 2025 May 27;20(5):e0322039. doi: 10.1371/journal.pone.0322039 (PMC12112190; doi:10.1371/journal.pone.0322039)
Supplement: Attachment — Submitted filename: Revision3_Response to the editor_ GebStart-tool.pdf [file pone.0322039.s012.pdf]

Response to the editor, revision 3

Comment	Responses/adaptations
1. Expand the discussion on the tool's clinical utility.	Thank you for this important comment. We were happy to elaborate the paragraph about the clinical benefit, see page 30, lines 430 and following:<br>*"The GebStart tool holistically assesses women's conditions during early labour, considering not only physical discomfort but also well-being at home, women's confidence in giving birth, and handling contractions. This facilitates the identification of women who require professional support and no longer feel comfortable remaining at home, ensuring they receive appropriate care and are neither neglected nor dismissed. [1,10]. Conversely, women who are comfortable at home should be encouraged to remain there for as long as possible, as later hospital admission has been associated with fewer interventions and improved obstetric outcomes [7,8]. This also enables the targeted use of resources for the women who need them [12]. The GebStart tool has the potential to support shared decision-making on the best place for each woman to stay. This is important for improving the provision of early labour care, but also women's experiences."*
2. Emphasize the preliminary nature of the findings due to sample size limitations.	Many thanks for this comment. We thoroughly revised the whole manuscript to make sure that the preliminary nature is clear. We especially specified this in the limitations and the conclusion.
